# The Evolving Role of Artificial Intelligence in Andrological Surgery: Current Landscape and Future Direction

**DOI:** 10.3390/jcm15041473

**Published:** 2026-02-13

**Authors:** Antonio Andrea Grosso, Francesca Conte, Luca Mazzola, Francesco Lupo Conte, Beatrice Giustozzi, Riccardo Ferretti, Marco Saladino, Daniele Paganelli, Luca Lambertini, Fabrizio Di Maida, Mattia Lo Re, Valeria Pizziconi, Gianni Vittori, Rino Oriti, Andrea Cocci, Andrea Mari, Andrea Minervini

**Affiliations:** Unit of Oncologic Minimally-Invasive Urology and Andrology, Department of Experimental and Clinical Medicine, University of Florence, Careggi Hospital, Largo Giovanni Brambilla 3, 50141 Florence, Italy; francesca.conte1@unifi.it (F.C.); luca.mazzola@unifi.it (L.M.); francescolupo.conte@unifi.it (F.L.C.); beatrice.giustozzi@unifi.it (B.G.); riccardo.ferretti@unifi.it (R.F.); marco.saladino@unifi.it (M.S.); daniele.paganelli@unifi.it (D.P.); luca.lambertini@unifi.it (L.L.); fabrizio.dimaida@unifi.it (F.D.M.); mattia.lore@unifi.it (M.L.R.); valeria.pizziconi@unifi.it (V.P.); giani.vittori@unifi.it (G.V.); rino.oriti@unifi.it (R.O.); andrea.cocci@unifi.it (A.C.); andrea.mari@unifi.it (A.M.); andrea.minervini@unifi.it (A.M.)

**Keywords:** artificial intelligence, erectile dysfunction, male infertility, microsurgery, robotic-assisted surgery

## Abstract

**Background**: With the rapid advancement of artificial intelligence (AI), its applications in andrology are expanding across diagnostic assessment, preoperative planning, intraoperative assistance, and postoperative management. This narrative review aims to synthesize current evidence regarding AI applications across the spectrum of andrological surgery. **Methods**: A comprehensive literature search was conducted using the PubMed, Scopus and Web of Science databases to identify relevant studies published between January 2020 and October 2025. The search strategy utilized combinations of keywords including “artificial intelligence,” “andrology,” “erectile dysfunction,” “male infertility,” “microsurgery,” and “robotic-assisted surgery.” Original research and review articles published in English were selected based on their clinical relevance to surgical practice. **Results**: AI has shown promise in the evaluation and management of erectile dysfunction (ED), male infertility-related microsurgery, and complex reconstructive procedures. AI-based models can improve risk prediction and diagnosis of ED, standardize semen analysis, support individualized selection of surgical candidates for varicocele repair and other interventions, and augment microsurgery through enhanced visualization and decision support. In the postoperative phase, AI-driven tools are being explored for complication prediction, functional recovery monitoring, and long-term quality-of-life follow-up, enabling more patient-centered, continuous care. **Conclusions**: AI holds significant promise for advancing precision medicine in andrological surgery by enhancing objective assessment and intraoperative guidance. However, large-scale, standardized datasets and rigorous multi-institutional validation are needed. Establishing robust ethical and legal frameworks will be essential to ensure the safe and effective integration of AI into routine andrological care.

## 1. Introduction

Andrological surgery spans multiple subspecialties, including procedures for erectile dysfunction (ED; e.g., penile prosthesis implantation and nerve-sparing interventions), male infertility-related microsurgery (e.g., microsurgical varicocelectomy, micro-TESE, vaso-vasostomy), penile deformity correction and reconstruction, and testicular and epididymal surgery. These procedures are technically demanding, often centered on fine anatomical structures such as neurovascular bundles, spermatic microvasculature, and intricate penile tissues, and have profound implications for sexual function, fertility, body image, and psychological well-being. Consequently, they impose particularly high requirements on surgical precision, functional preservation, and individualized decision-making. In traditional practice, many key steps in andrological surgery rely heavily on operator experience and subjective assessment, for example, patient-reported ED severity, manual microscopic semen analysis, intraoperative identification and preservation of neurovascular structures, and qualitative evaluation of penile deformity. AI, through machine learning (ML), deep learning (DL), computer vision, and natural language processing (NLP), provides data-driven, objective tools across these domains, and has therefore rapidly gained traction in andrology and reproductive medicine [1,2,3,4]. Compared with oncologic urology, where AI has been extensively applied to imaging segmentation, 3-dimensional (3D) reconstruction, and robotic path planning, research on AI in andrological surgery started later but has grown rapidly in recent years [1,5,6,7].

Current areas of application include AI-based risk prediction and diagnosis, AI-assisted 3D modeling and navigation in reconstructive surgery, longitudinal management such as personalized rehabilitation pathways, and long-term quality-of-life (QoL) monitoring. This review focuses specifically on AI in andrological surgery, re-organizing and synthesizing available evidence around surgical indications and workflow stages. We detail technical approaches, application scenarios, and supporting clinical data, and we discuss challenges and future directions for integrating AI into andrological practice.

## 2. Materials and Methods

We conducted a search for literature addressing the application of AI in andrological surgery. Relevant studies published between January 2020 and December 2025 were identified through searches of PubMed, Scopus and Web of Science. The search strategy incorporated key terms related to AI technologies and andrological surgical practice, including “artificial intelligence,” “andrology,” “erectile dysfunction,” “male infertility,” “microsurgery,” “robotic-assisted surgery,” and “postoperative management.” These terms were used in various combinations to capture a broad yet clinically relevant spectrum of publications. To facilitate accurate interpretation and critical appraisal, the review was limited to articles published in English. Given the rapidly evolving nature of AI-related technologies, particular emphasis was placed on original studies and review articles published within the most recent three years. Study selection was guided by clinical relevance; methodological quality; and potential implications for surgical decision-making, perioperative management, and outcomes in andrological practice (Table 1). A narrative design was opted to present the results.

## 3. Results

### 3.1. The Intersection of Andrological Specialties and Artificial Intelligence

**Figure 1:**
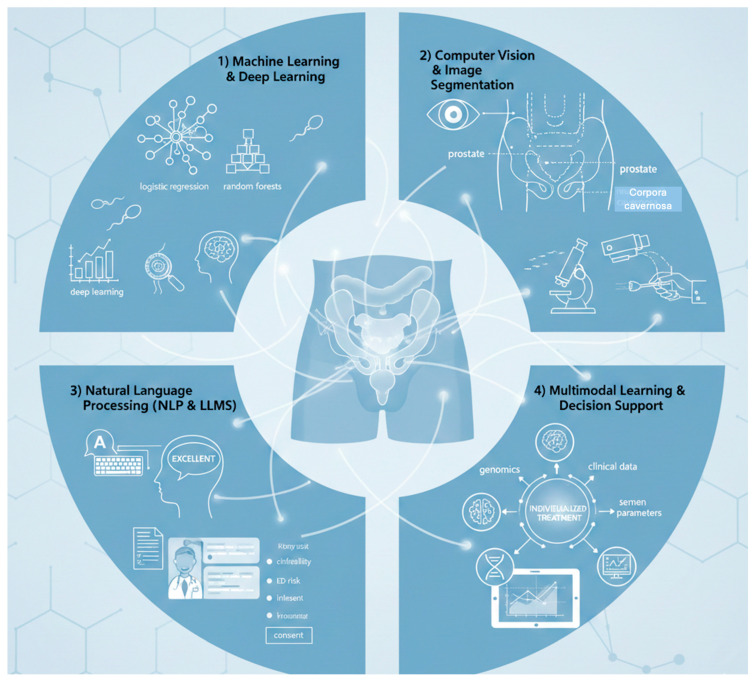
AI applications in andrological surgery.

AI applications in andrological surgery primarily build on four technical pillars (Figure 1):

(1) Machine learning and deep learning. Conventional machine-learning approaches, such as logistic regression, random forests and support vector machines, have been widely used to develop prediction models for ED risk, treatment response in male infertility, and postoperative complications [5,8]. More recently, deep-learning architectures, particularly convolutional neural networks (CNNs) and 3D U-Net derivatives, have enabled automated sperm detection and classification in microscopy, segmentation of neurovascular bundles and erectile tissue on magnetic resonance imaging (MRI), and real-time recognition of relevant structures in surgical videos [9,10,11].
(2) Computer vision and image segmentation. High-resolution, standardized image analysis is central to many andrological procedures. In semen microscopy, DL supports automated sperm counting, motility assessment, and morphology classification. In pelvic MRI, DL-based segmentation can delineate the prostate, neurovascular bundles, internal pudendal arteries, and corpora cavernosa, producing precise maps of ED-relevant anatomy to support nerve-sparing surgery and radiotherapy planning [10,12]. In addition, computer-vision methods applied to operative videos can identify anatomical landmarks and track instruments, with potential value for microsurgical training and intraoperative guidance.
(3) Natural language processing (NLP) and large language models (LLMs). NLP and LLMs have been used to automatically summarize clinical notes and extract structured variables related to ED and infertility but also for patient education and informed consent. In particular, in counseling for penile prosthesis surgery, expert reviewers rated AI-generated responses to common patient questions as “excellent” in more than 70% of cases [13]. In addition, conversational agents can support semi-automated follow-up and symptom triage in routine care pathways [7,14].
(4) Multimodal learning and decision support. Andrological decision-making frequently integrates diverse data sources (clinical, hormonal, semen parameters, imaging, questionnaires, and sometimes genomics). Multimodal AI models are well-suited to capture high-dimensional patterns across these layers, supporting more robust risk stratification and individualized treatment recommendations [1,4,6].

An overall view of the included studies is depicted in Table 2.

### 3.2. AI Applications in Erectile Dysfunction-Related Surgery

Recently, several studies have developed ML-based ED risk prediction models using large population datasets [5,8]. Using data from NHANES 2001–2004, Chen et al. constructed ML models incorporating age, body mass index, lifestyle variables, and cardiovascular risk factors to predict ED. The best-performing model achieved an area under the receiver operating characteristic curve above 0.88 [5]. Feature importance analysis confirmed age, obesity, and cardiovascular risk as major predictors, but ML approaches better captured non-linear relationships and variable interactions than traditional regression models. Such models could support population-level screening, early intervention, and preoperative baseline assessment of erectile function. More advanced models integrating questionnaires, laboratory markers, and imaging are being explored and may provide richer phenotyping of ED subtypes. In addition, NLP methods can process unstructured clinical narratives and questionnaires to automatically extract relevant features, improving workflow efficiency and consistency in ED assessment [7,14].

### 3.3. AI-Enabled Neuroanatomical Mapping and Nerve Protection

In surgeries that threaten ED, such as radical prostatectomy, pelvic tumor surgery, and pelvic fracture repair, AI is increasingly used for automatic contouring of targets and critical structures. Although much of this work originates in radiation oncology and pelvic surgery, the methodology is directly relevant to andrological functional preservation. Schubert et al. used dedicated pelvic MRI and DL-based models to automatically contour prostate targets and neurovascular structures in MR-guided radiotherapy, achieving surface dice-similarity coefficients up to ~0.8 compared with expert manual segmentations [10]. In radiotherapy planning for localized prostate cancer, Balagopal et al. developed a DL model for automatic segmentation of the internal pudendal artery on CT, improving consistency and efficiency in contouring radiation-sensitive structures relevant to erectile function [12]. Together, these methodologies establish an anatomical and computational framework for optimizing several urological interventions. By integrating patient-specific data, these approaches enable refining strategies for nerve-sparing or nerve-grafting during surgeries associated with ED, targeting dose-contouring to protect critical erectile structures from radiation damage and enhancing preoperative mapping and facilitating AR guidance for penile prosthesis implantation or complex reconstructive procedures.

### 3.4. AI in Penile Prosthesis and Other ED Procedures

AI has not yet been fully integrated into the operative control of penile prosthesis implantation but is emerging in several supporting roles. Shayegh et al. evaluated a publicly available LLM (ChatGPT) in responding to common patient questions about inflatable penile prostheses. Over 70% of AI-generated responses were rated “excellent” by experts in terms of structure, evidence-based content, and clarity [13]. Such tools can improve pre-visit education, enhance patient understanding, and reduce repetitive counseling workload for surgeons. AI-enabled follow-up platforms and NLP-driven analysis of patient reports can flag high-risk signals such as pain escalation, signs of infection, mechanical malfunction, or dissatisfaction. Although current evidence is preliminary and often small-scale, these systems may support earlier intervention and more structured long-term monitoring. An emerging scenario is related to digital twin and virtual planning. In fact, with high-resolution MRI and 3D reconstruction, a “penile–pelvic digital twin” could be created, onto which AI-derived models might simulate different implant sizes, positions, and configurations. This may be particularly valuable for complex deformities or revision cases, potentially improving functional and cosmetic outcomes. This concept remains largely exploratory.

### 3.5. AI in Male Infertility-Related Surgery

Semen analysis remains the cornerstone of male infertility evaluation but is limited by labor-intensive manual microscopy, poor inter-observer reproducibility, and variable adherence to guidelines. AI-based computer-assisted semen analysis (CASA) systems use ML and DL algorithms to perform automated and standardized assessment of sperm concentration, motility, and morphology [9,16,17]. Recent studies report that AI systems can reach accuracies of ~88% for morphology assessment and ~90% for motility evaluation [9]. Using high-magnification microscopic images, DL models can also extract fine-grained features to identify head, neck, and tail abnormalities and distinguish live from dead sperm, enabling more detailed morphological profiling [16]. In parallel, exploratory research is assessing whether AI can help evaluate deeper quality indicators such as the DNA fragmentation index, although this remains largely methodological at present [18]. In the future, by integrating semen findings with hormonal profiles, genetic information, and other omics data, AI may generate a more comprehensive “sperm health score,” which could inform both prognostication and the selection of surgical versus assisted reproductive interventions. An important direction for AI in male infertility surgery is the prediction of benefit from specific interventions, particularly varicocele repair. By integrating age, baseline semen parameters, hormone levels, ultrasound features, and clinical history, ML models, such as random forests, can be trained to predict whether postoperative semen parameters will significantly improve [19]. In a multi-institutional analysis, Ory et al. used ML models to predict “sperm parameter upgrading” after varicocele repair, demonstrating improved discrimination compared with conventional statistics [19]. Clinically, such models could enable more personalized counseling by identifying patients most likely to benefit from surgery, helping avoid interventions with a low likelihood of improving semen parameters or fertility, and informing the timing and selection of adjunctive assisted reproduction strategies. Conceptually similar approaches could be extended to vaso-vasostomy, epididymal sperm aspiration, and other obstructive infertility procedures, integrating etiology, obstruction duration, partner fertility, and ART feasibility into a unified decision framework [22,23]. Experimental systems combine high-resolution microscopic imaging with DL-based image analysis to identify seminiferous tubules more likely to contain sperm during micro-TESE, theoretically reducing random sampling and tissue damage [23]. Evidence for improved sperm retrieval rates or reduced complications is not yet available, but potential benefits include shorter operative times and more targeted tissue sampling. For complex spermatic cord or scrotal anatomy (e.g., anatomical variants, post-surgical scarring), AI-driven 3D reconstruction and virtual/augmented reality (VR/AR) can provide preoperative roadmaps and potential intraoperative overlays [15]. Automatically segmented spermatic vessels, vas deferens, and adjacent structures could be projected onto the live surgical field, theoretically minimizing inadvertent injury.

### 3.6. AI in Penile Deformity Correction and Reconstruction

AI-supported automatic segmentation and 3D modeling, based on preoperative MRI or high-resolution ultrasound, can generate patient-specific anatomical models of the penis and surrounding structures. Using DL, these systems can delineate soft-tissue boundaries, including the corpora cavernosa, corpus spongiosum, urethra, and key vessels and nerves; quantify the degree and axis of curvature in Peyronie’s disease or congenital curvature; and potentially integrate the resulting models into augmented-reality (AR) platforms to overlay preoperative anatomy onto the intraoperative view [1]. Clinically, such tools could facilitate accurate measurement and planning of application or grafting approaches, improve implant or flap planning in complex penile reconstruction (e.g., after failed hypospadias repair, oncologic resection, or trauma), and help anticipate risks to erectile tissue integrity and vascular supply. Although dedicated clinical studies on AR-guided penile reconstruction are currently limited, encouraging results from AR/MR applications in urologic oncology surgery provide a strong methodological foundation for expansion into penile surgery.

### 3.7. Testicular and Epididymal Surgery

AI applications in testicular tumor and epididymal surgery remain at an early stage, but several promising directions have been proposed [7,24]. ML models that combine scrotal ultrasound, MRI, and serum tumor markers may improve discrimination between benign and malignant lesions and help predict stage, thereby refining selection for organ-sparing surgery versus radical orchiectomy. In parallel, AI-driven analysis of intraoperative spectral or real-time imaging could move toward “instant pathology,” supporting margin assessment and intraoperative decision-making during testis-sparing procedures. Finally, AI-assisted identification of critical ducts and vascular structures during epididymal surgery may help standardize fertility-preserving techniques and reduce iatrogenic injury. Evidence to date is largely conceptual or limited to cross-sectional studies, but as standardized andrology imaging datasets with expert annotations become available, this domain may evolve rapidly.

### 3.8. AI in Postoperative Management, Rehabilitation, and Long-Term Follow-Up

Postoperative complications in andrological surgery, such as infection, hematoma, mechanical failure (for prostheses), persistent pain, or failure to achieve expected sexual or reproductive outcomes, can substantially affect patient satisfaction. AI-based risk prediction models established in other urologic procedures [4,25] offer a template for adaptation to andrological surgery, with specific target outcomes such as prosthesis-related infection and reoperation, lack of improvement or worsening of erectile dysfunction after surgery, and inadequate gains in semen parameters or pregnancy rates following infertility surgery. By integrating demographic factors, baseline functional status, surgical technique, operative time, blood loss, and early postoperative parameters, ML models can stratify individual risk and guide intensity of monitoring and prophylactic measures.

AI can support personalized rehabilitation and remote monitoring. In detail, by learning from longitudinal data on functional recovery trajectories (e.g., erectile function scores, ejaculation patterns, semen parameters), psychological status, and QoL measures across cohorts, AI models could predict individualized recovery curves for new patients. Such predictions may help optimize the timing and intensity of penile rehabilitation after prosthesis implantation or nerve-sparing surgery, personalize follow-up semen testing schedules after varicocele repair or vasovasostomy, and identify patients at high risk of delayed or incomplete recovery, thereby prompting earlier psychological, behavioral, or supportive interventions. Moreover, mobile applications and web-based platforms, combined with NLP and automated questionnaire analysis, can structure patient-reported information and triage alerts. High-risk signals, such as worsening pain, wound or urinary symptoms suggestive of infection, or significant decline in sexual function, can be flagged for clinician review [7,20]. This is particularly valuable for patients with geographic or logistical barriers to in-person follow-up. AI-assisted analysis of patient-reported outcome measures (PROMs) could link early recovery metrics, such as the Quality of Recovery-15 (QoR-15), to mid- and long-term QoL, consistent with findings from other surgical fields where 24 h QoR-15 scores correlated with 3-month QoL outcomes [21]. These approaches may also help identify modifiable drivers of long-term satisfaction, including expectation management and the quality of perioperative counseling, thereby strengthening preoperative education and shared decision-making. In addition, clustering and trajectory modeling can characterize heterogeneous recovery patterns across patients, supporting more individualized rehabilitation strategies. As andrology-specific PROMs and long-term datasets accumulate, AI-enabled analyses may become integral to patient-centered outcome research and service planning.

## 4. Challenges, Ethics, and Legal Considerations

The development of robust AI systems in andrology is limited by the scarcity of large, high-quality, expert-annotated datasets, particularly for microsurgery and operative videos, along with substantial heterogeneity in data standards across centers and devices. In addition, erectile dysfunction and infertility datasets may embed demographic and socio-cultural biases, which can compromise model generalizability and equity if not explicitly assessed and mitigated [26,27,28,29]. Moreover, semen analyses, sexual function assessments, and fertility data are highly sensitive, raising stringent requirements for data anonymization, secure storage, and governed sharing. Approaches such as de-identification, federated learning, and blockchain-based data management have been proposed to reconcile data utility with privacy and security [27,28,29].

Most AI studies in andrological contexts are single-center and retrospective, with limited external validation and uncertain generalizability. Performance often drops when models are tested on data from different institutions, imaging protocols, or patient populations [30,31,32]. Moving beyond proof of concept will require multicenter, multi-ethnic, large-scale prospective studies; consistent adherence to standardized AI reporting guidelines [31]; and validation frameworks that assess not only technical performance (e.g., accuracy and robustness) but also clinical effectiveness, including effects on patient outcomes and workflow [30].

AI can enhance objectivity and efficiency in ED and infertility diagnosis and treatment but introduces “black-box” decision risks and blurred responsibility. Clinicians may increasingly act as supervisors of AI-generated suggestions rather than sole decision-makers [1,33]. In this setting, clinicians must retain critical judgment and avoid over-reliance on AI, patients should be explicitly informed when AI is used in their care along with its limitations and uncertainties, and legal frameworks should more clearly define how responsibility is shared among clinicians, healthcare institutions, and developers when AI-influenced decisions contribute to adverse outcomes. Ethics consultation frameworks are being adapted to address AI-specific dilemmas in clinical practice [33].

## 5. Conclusions

Future priorities include: (1) Building high-quality, standardized AI datasets of images, pelvic MRI, operative videos, and longitudinal PROMs. These are essential to drive algorithm development and validation. (2) Integrating imaging, clinical, and multi-omics data in order to shift from “anatomy–function” to “anatomy–function–molecular” decision-making, opening new avenues for precision andrological surgery. (3) Developing explainable, clinically usable AI systems to enhance clinician and patient trust and facilitate clinical adoption. (4) Accelerating clinical validation and guideline integration through high-quality prospective studies and, where feasible, randomized controlled trials.

In summary, AI offers unprecedented opportunities to improve precision, safety, and personalization in andrological surgery while posing significant technical, ethical, and legal challenges. Through multidisciplinary collaboration, rigorous data governance, and robust clinical validation, AI can be safely and effectively integrated into andrological practice, ultimately improving outcomes and quality of life for male patients and their partners.

## Figures and Tables

**Table 1:** Search strategy.

Items	
Date of Search	December 2025
Databases and other sources	PubMed; Scopus; Web of Science
Search-terms used	“artificial intelligence,” “andrology,” “erectile dysfunction,” “male infertility,” “microsurgery,” “robotic-assisted surgery,” and “postoperative management”
Timeframe	January 2020–December 2025
Inclusion criteria	Original Studies and Review articles; Articles published in English; Studies with direct clinical relevance to andrological surgery
Selection process	The literature screening was conducted by two researchers (A.A.G. and F.C.). If there was debate on literature inclusion, it was resolved through discussion; if no consensus could be reached, a third senior researcher (L.M.) was consulted for arbitration. Given the rapidly evolving nature of Al- related technologies, particular priority was given to original studies and review articles published within the most recent three years (2023–2025).

**Table 2:** Summary of the included studies.

Study Domain	Key Study/Author	AI Model Type	Primary Clinical Outcome
**Erectile disfunction & Penile surgery**	Chen et al. [5]	Machine Learning (ML)	Achieved AUC > 0.88 in predicting erectile dysfunction risk.
	Qin et al. [7] Shayegh et al. [13] Khawaja et al. [14]	Natural language processing (NLP) and large language models (LLMs)	Improving workflow efficiency and consistency in ED assessment
	Schubert et al. [10] Balagopal et al. [12] Silva et al. [15]	Deep Learning (3D U-Net)	Improving anatomical knowledge and preoperative surgical planning
**Semen Analysis**	Ghayda et al. [9] Chen et al. [16] Richie et al. [17] Nussinov et al. [18] Ory et al. [19]	Deep Learning (CNNs)	Accuracy for morphology and sperm motility assessment. Improving the prediction of fertility
**Patient Education**	Shayegh et al. [13] Hirtsiefer et al. [20] Le Bescond et al. [21]	Large Language Model (ChatGPT-4)	Support patient decision-making

## Data Availability

No new data were created in this study. Data sharing is not applicable to this article.

## Abbreviations

The following abbreviations are used in this manuscript:

AI	Artificial Intelligence
ED	Erectile Dysfunction
ML	Machine Learning
DL	Deep Learning
NLP	Natural Language Processing
3D	3 Dimensional
QoL	Quality of Life
CNNs	Convolutional Neural Networks
MRI	Magnetic Resonance Imaging
LLMs	Large Language Models
CASA	Computer-Assisted Semes Analysis
VR/AR	Virtual/Augmented Reality
AR	Augmented Reality
PROMs	Patient-Reported Outcome Measures
QoR-15	Quality of Recovery-15
